# Eco-Innovative UHPC—Enhancing Sustainability, Workability, and Ductility with Recycled Glass Cullet Powder and Plastic Bottle Hybrid Fibers

**DOI:** 10.3390/ma17020393

**Published:** 2024-01-12

**Authors:** Mohammad Iqbal Khan, Galal Fares, Yassir M. Abbas, Fahad K. Alqahtani

**Affiliations:** Department of Civil Engineering, College of Engineering, King Saud University, P.O. Box 800, Riyadh 11421, Saudi Arabia; galfares@ksu.edu.sa (G.F.); yabbas@ksu.edu.sa (Y.M.A.); bfahad@ksu.edu.sa (F.K.A.)

**Keywords:** glass cullet, mechanical strength, plastic bottle fibers, slump–flow, recycling hazardous waste materials, ultra-high-performance concrete (UHPC)

## Abstract

Utilizing waste materials in producing ultra-high-performance concrete (UHPC) represents a highly effective approach to creating environmentally sustainable concrete using renewable resources. This study focused on incorporating ground glass cullet (GP) at various replacement levels in UHPC production. Additionally, plastic bottle fibers (PBFs) were derived from discarded plastic bottles and employed in the mix. The replacement levels for GP spanned from 0% to 40%. Single-use plastic bottles were transformed into strip fibers, both with and without the inclusion of microsteel fibers, at varying contents of 1.1% and 2.2% (volume-based). A single-fiber test was conducted on PBFs under different strain rates. The introduction of optimal GP content had a profound positive iMPact on compressive strength. Incorporating 2.2% plastic strips induced strain hardening behavior, while further inclusion of microsteel fibers resulted in substantial enhancements in mechanical properties. Two types of microsteel fibers were employed, characterized by different aspect ratios of 65 and 100. The optimum GP content was identified as 10%. Moreover, the UHPC mix achieved superior compressive strength, exceeding 140 MPa when composed of 1.3% (volume-based) microsteel fibers with an aspect ratio of 65 and 2.2% PBF (volume-based). Notably, mixtures featuring microsteel fibers with a higher aspect ratio demonstrated the highest flexural strength, exceeding 8000 N in the presence of 2.2% PBF. Longer microsteel fibers exhibited adequate slip properties, facilitating strain transfer and achieving a strain-hardening response in conjunction with plastic bottle fibers. These findings illuminate the potential for harnessing hazardous waste materials to improve the performance and sustainability of UHPC formulations.

## 1. Introduction

### 1.1. Background

The accumulation of waste all over the world is a standing environmental issue to be solved in an environmentally friendly manner. The sustainability concept relies on renewable resources and management. Renewable waste materials should be well used as eco-friendly resources for various applications, especially those valid for construction. The advent of the 21st century has seen humanity grappling with the increasingly complex issues of waste accumulation and its subsequent management. Originating from a diverse array of sectors, including domestic residences, commercial enterprises, educational facilities, gastronomic establishments, and agricultural ventures, waste has become an omnipresent byproduct of human endeavor. According to quantifiable metrics, the global waste volume stood at 2.02 billion tons in 2016. Forecasts indicate a troubling trajectory, with projections estimating an increase to 2.59 billion tons by 2030, culminating in a staggering 3.4 billion tons by the mid-century [1]. Global authorities on sustainability have drawn attention to the alarming annual accumulation of 209 million tons of post-consumer glass cullet [2,3]. Many studies (e.g., [4,5,6]) have examined this unused resource. For example, the recycling of waste glass in France has been advanced to recycling seven glass bottles out of 10 [7]. These studies have led researchers from different fields to consider how it could be used. In parallel, the recycling of thermoplastic water bottles made of polyethylene terephthalate (PET) has also been the subject of considerable research [8,9,10]. The formulation of lightweight aggregates using dredging sediments and different types of plastic waste was successful and conformed to European standards [11]. While glass recycling often involves mechanical processes like grinding, PET recycling typically employs thermal methods [12,13]. The material is heated and stretched into fibers, which are used in various applications [14,15,16]. These fibers have found utility in textiles [17], insulation [18], and even as reinforcement in polymer composites [19]. Given its substantial consumption of raw materials and corresponding high levels of waste generation, the building and construction sector presents an ideal candidate for incorporating these recycled materials. Over 53 Mt of waste are produced annually in Saudi Arabia [20], where plastic waste represents 5–17% of the total solid waste and glass waste represents 3–5% of this amount.

### 1.2. Bibliographical Overview

In the scholarly literature, researchers (e.g., [21,22,23]) have intensely examined alternative materials, such as ground post-consumer glass, that can be added to cement to make it more sustainable. The main challenge with conventional concrete is related to its durability and ductility issues, which can be improved using the current concrete technology to improve its properties using recycled waste materials with cementitious properties. This ground glass type has emerged as a viable alternative to traditional additives in cement (supplementary cementation material (SCM)). Scholarly articles, including key studies by Borges et al. [24] and Wang et al. in [25], have confirmed that these glass additives interact well chemically and mechanically with cement, making them a feasible choice for creating strong and durable concrete. The efficacy of this approach is primarily attributed to transforming glass waste into glass powder (GP) [26,27,28]. The advent of GP as an SCM is not merely a matter of waste utilization but also a way to avoid the well-documented drawbacks of using glass as an aggregate in concrete, primarily the risk of alkali–silica reactivity [29,30,31]. An unmanaged chemical reaction of this sort poses a severe risk to the stability of concrete structures [31,32,33]. Transforming waste glass into GP serves a dual purpose: it mitigates waste disposal concerns and resolves a critical engineering problem.

In a valuable investigation, Zhao et al. [34] advanced the understanding of how GP functions as a pozzolanic material. The investigators conclusively showed that GP effectively strengthens the bond between new and recycled concrete aggregates, improving a key area often considered the weak link in composite materials [35]. Notably, the researchers also explored new ground by assessing GP’s effectiveness when replacing up to 20% of traditional materials, discovering the formation of intricate calcium–silicate–hydrate (C–S–H) formations on the glass particles. This work aligns with the findings of Dobiszewska et al. [32], solidifying a shared scientific perspective on the matter.

Moreover, a study by Frigione [36] offered a groundbreaking perspective by investigating the use of PET as a replacement for fine aggregates in concrete mixes. This research revealed an interesting trade-off: while the workability of the concrete improved remarkably, there was an insignificant decline in the compressive strength. However, the material exhibited improved ductility, a property often ignored but crucial in specific structural applications. For instance, this includes enhancing the load-carrying capacity of established infrastructures and those influenced by seismic activity [37,38,39]. Similarly, Umasabor and Daniel [40] explored the effects of PET aggregate replacement levels ranging from 5 to 15%. Their findings suggested that the optimal replacement level was 5%, beyond which the material’s flexural strength started to reduce. The literature robustly covers the use of ground post-consumer glass and PET as alternative materials in concrete, detailing their mechanical and chemical properties. However, there is a notable absence of data on their environmental iMPact, economic viability, and long-term structural integrity. Most significantly, limited studies have examined the hybrid combination of GP and PET fibers, presenting an open avenue for future research.

### 1.3. Scope, Objectives, and IMPact of the Current Research

This research aims to merge two related but often separately studied fields, the application of GP and thermally processed PET fibers (denoted here as PBF), in developing construction materials. The goal is to develop an ultra-high-performance concrete (UHPC) mix, containing GP and PBF, that meets modern engineering standards. A unique feature of this approach is the inclusion of specialized microsteel fibers, chosen to enhance the mechanical benefits. This comprehensive strategy improves the mechanical properties of the resulting material and enhances its mechanical behavior under different stress conditions. This research offers an advanced and multipurpose concrete mix that could set new industry standards for material quality, including strength, durability, and ductility. Notably, the new composite meets the strict mechanical strength and durability criteria required in today’s construction projects. Thus, the material is more than a theoretical concept; it has practical applications in various construction settings, ranging from major infrastructures to commercial and residential buildings. Further, this study promotes sustainable practices by converting waste materials into valuable construction resources, aligning with broader environmental objectives. Therefore, this research is an innovative step toward evolving sustainable construction practices. The current approach to recycling glass powder and plastic bottle hybrid fibers in UHPC mixes presents a great potential for the sustainable recycling of waste in various applications related to the UHPC domain.

## 2. Materials and Methods

### 2.1. Materials

In the present study, we investigated not only the primary materials of interest (i.e., ground glass powder (GP) and bisphenol F (BPF)) but also other conventional SCMs (fly ash (FA) and silica fume (SF)). Here, desert dune sand (DS) served as the fine aggregate. To investigate these materials’ chemical and mineralogical compositions, we employed a range of analytical techniques, including X-ray diffraction (XRD) using an Empyrean instrument and X-ray fluorescence (XRF) performed on an AXIOS mAX system. These instruments are products of PANalytical (Almelo, The Netherlands). Additionally, the particle size distribution for each fine powder was assessed utilizing an LA-950V2 particle size distribution (PSD) analyzer (Horiba, Kyoto, Japan). Furthermore, microstructural analyses were carried out using a Versa 3D dual-beam scanning electron microscope (FEI, Amsterdam, The Netherlands). The subsequent section outlines the methods of preparation and the characteristics of the materials utilized in the current study.

#### 2.1.1. Glass Powder

**Figure 1:**
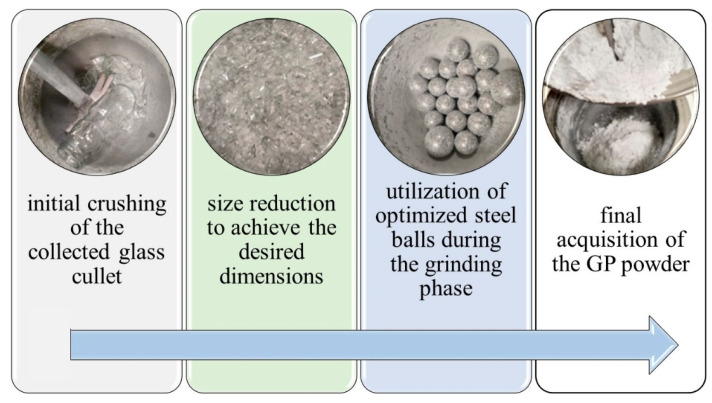
Refining glass cullet into fine GP by a special grinding technique.

**Figure 2:**
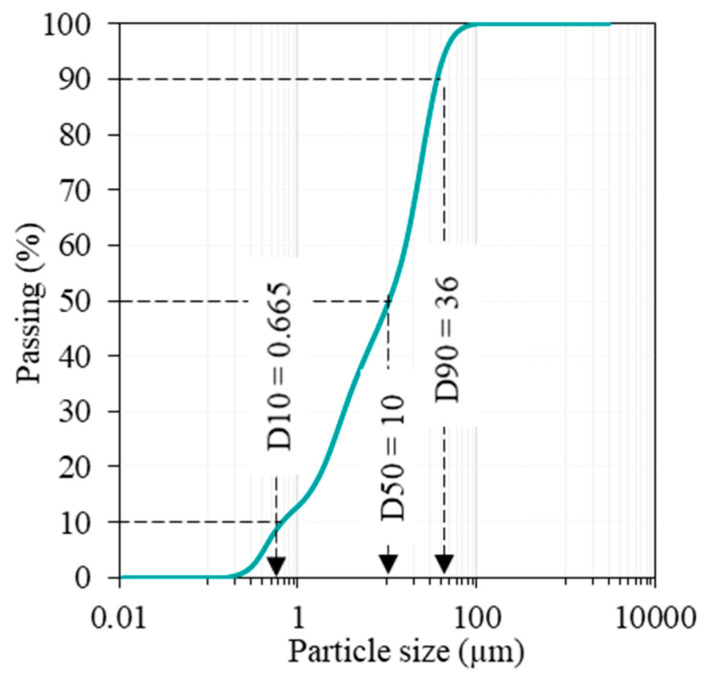
Particle size distribution analysis of GP.

In this study, for the preparation of GP and BPF, post-consumer glass bottles were collected, crushed, and ground to achieve a median particle size (D50), approximating that of cement (around 10 µm), as illustrated in Figure 1. The target D50 of the produced powder was achieved, as confirmed in Figure 2.

#### 2.1.2. Plastic Bottle Fibers (PBFs)

**Figure 3:**
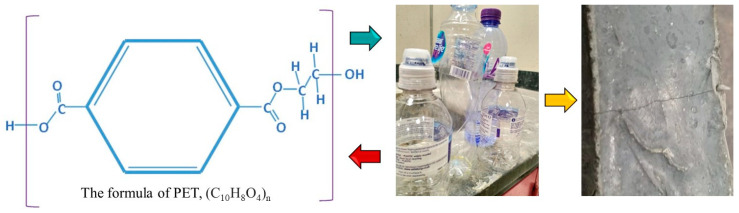
The chemical structure of PET utilized in the formulation of UHPC.

**Figure 4:**
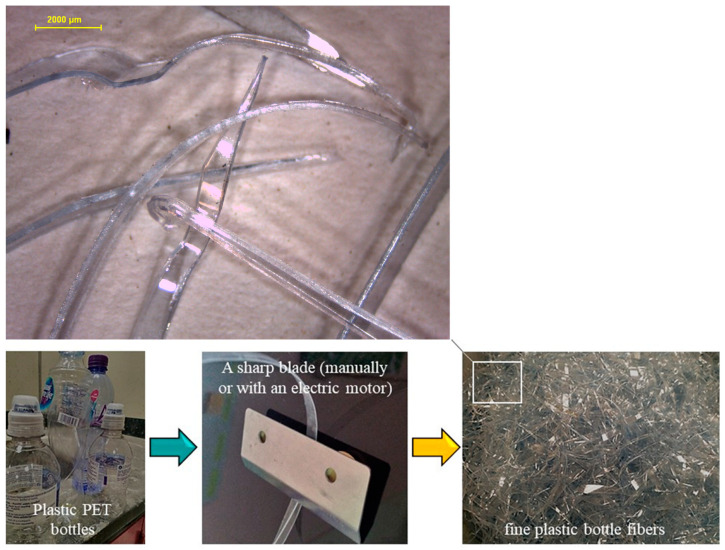
The procedure for acquiring PBF employed a sharp blade with the aspect ratio validated via optical microscopy.

In the present investigation, polyethylene terephthalate (PET), a predominant contributor to global plastic pollution, was chosen for detailed study and analysis. The chemical structure of the selected PET sample is depicted in Figure 3. The presence of polycarboxylate and hydroxyl functional groups enhances its adhesive interaction with cement paste. Employing precision-engineered blades set at a specific distance from a stationary surface, plastic bottles were effectively shredded into fine fibers with widths less than 500 µm and lengths ranging between 10 and 12 mm, as illustrated in Figure 4. This component is readily adaptable for incorporation into automated systems, facilitating broader applications and potential for industrial scale-up.

#### 2.1.3. Conventional Types of Cement

**Figure 5:**
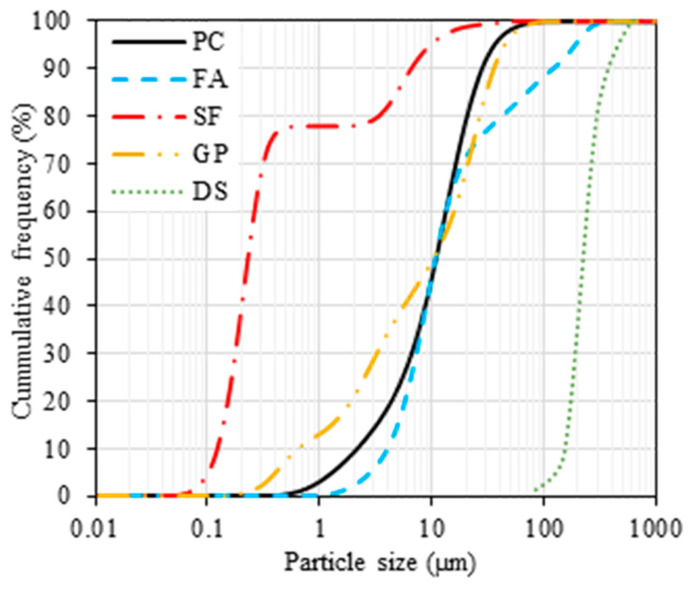
Particle size distribution analysis of fine powders.

**Figure 6:**
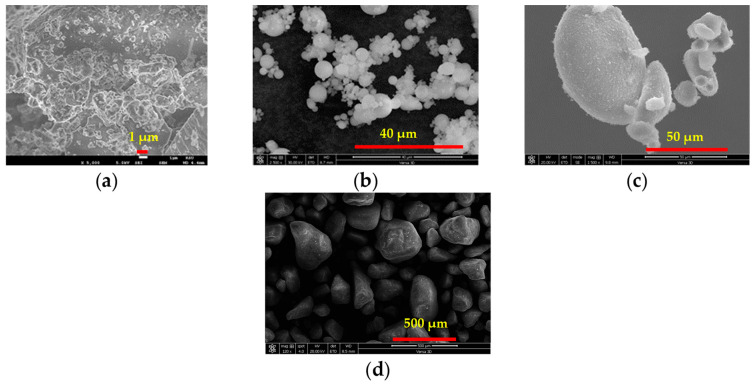
Photomicrographs of (**a**) GP, (**b**) FA, (**c**) SF, and (**d**) DS.

In this research, samples of Portland cement (PC), silica fume (SF), and Class F fly ash (FA) were sourced from a local vendor. Particle size distribution (PSD) analyses for these materials and DS are depicted in Figure 5. Table 1 presents a coMParative assessment of the physicochemical attributes of the GP, FA, and SF coMPared to PC. Scanning electron microscopy (SEM) images, illustrating the microstructures of GP, FA, and SF, are provided in Figure 6. Notably, the presence of characteristic cenospheres in the FA was validated, while a condensed form of SF was also observed.

#### 2.1.4. Natural Aggregates, Steel Fibers, and Chemical Admixtures 

**Figure 7:**
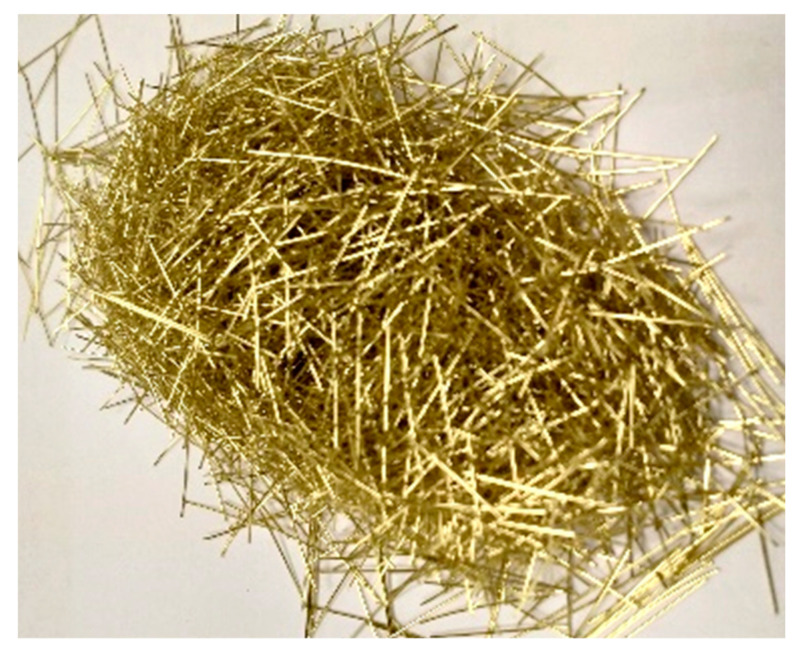
Microscopic examination of microsteel fibers (designated as SFib1).

**Figure 8:**
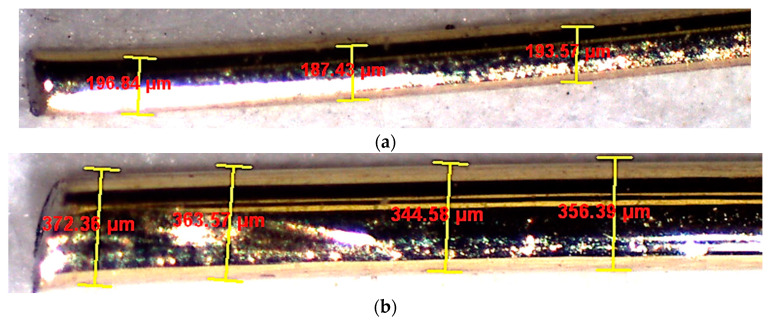
Measurement of microsteel fiber diameters of different steel size groups: (**a**) SFib1 and (**b**) SFib2.

In the current study, the physical characteristics of fine aggregates were quantified by a bulk specific gravity of 2.65, measured under saturated surface dry (SSD) conditions, accoMPanied by a water absorption rate of 0.3% and a fineness modulus of 2.67. For the formulation of the UHPC blends, two distinct categories of microsteel fibers were utilized. Notably, the authors previously conducted the optimization of these fibers in earlier research [41]. Microscopic analyses of these fibers are visually represented in Figure 7 and Figure 8. The physical properties of the microsteel fibers are listed in Table 2. The table summarizes the different sizes and aspect ratios of the employed steel fibers (designated as SFib1 and SFib2), which were collected and characterized. 

It is crucial to emphasize that the UHPC formulation incorporated a high-range water-reducing admixture composed of polycarboxylate ether-based substances, abbreviated as PCE. The inclusion of PCE enhanced the workability and flow characteristics of the concrete mix, facilitating easier placement and coMPaction without compromising the material’s mechanical properties.

### 2.2. Preparation and Testing Protocols

#### 2.2.1. Tensile Properties of PET

**Figure 9:**
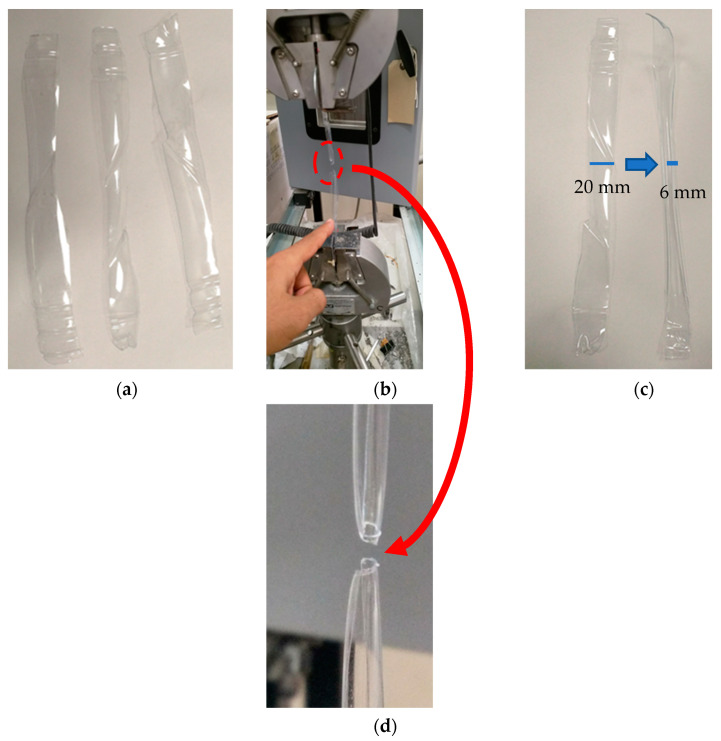
Test setup for (**a**) single plastic strip (20 mm × 120 mm) for the (**b**) pullout test and (**c**) coMParison between the prior and post-tension test tensile properties of the plastic strips and (**d**) ruptured strip to simulate fibers from plastic bottles.

To assess the tensile properties of the PET, test specimens were fabricated in dimensions of 20 × 120 mm and subjected to varying strain rates, as illustrated in Figure 9. 

#### 2.2.2. Formulation of the UHPC Mix

The main study methodology commenced with determining the target volume of the plastic fibers, followed by calculating the corresponding volume using the density of the plastic used. The optimized glass cullet powder was also defined through a series of optimization tests in the presence and absence of fine aggregates. Glass powder was to be homogenized with solid materials until a flowable cementitious matrix was prepared, followed by the addition of microsteel fibers under low-speed mixing, followed by high-speed mixing for 1 min, followed by the addition of plastic fibers and high-speed mixing for 30 s. The final mix was then to be cast and cured for testing.

**Figure 10:**
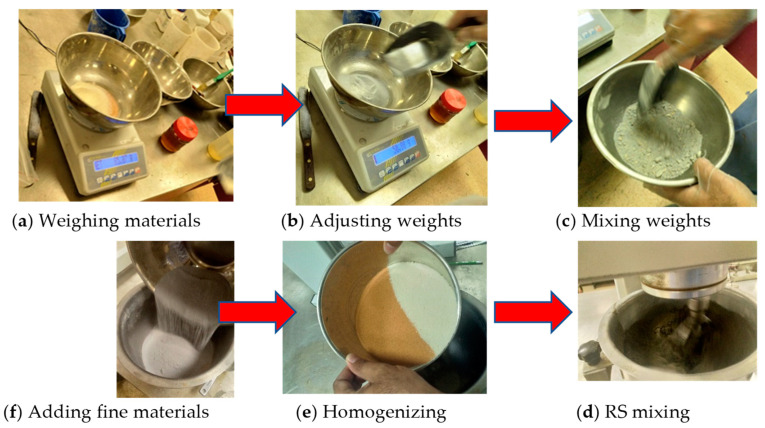

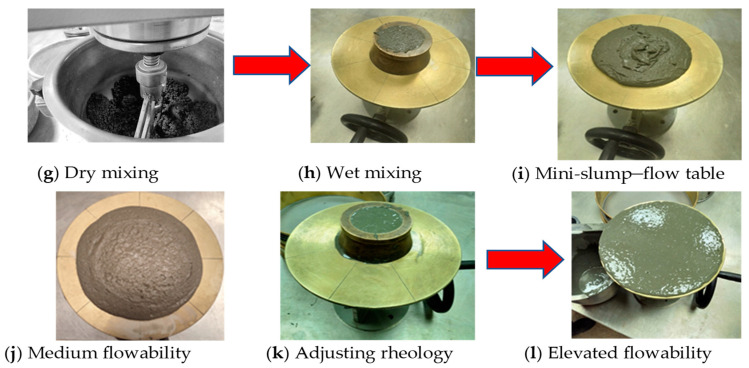
Pictorial steps to prepare the UHPC mixtures: (**a**) weighing materials, (**b**) adjusting weights, (**c**) mixing weights, (**f**) adding fine materials, (**e**) homogenizing, (**d**) RS mix, (**g**) dry mixing, (**h**) wet mixing, (**i**) mini-slump–flow table, (**j**) medium flowability, (**k**) adjusting rheology, and (**l**) elevated flowability.

A comprehensive depiction of the mix formulation is provided in Figure 10. The process commenced with precisely weighing constituents, followed by dry blending for uniformity. Subsequently, water, pre-mixed with an optimal quantity of superplasticizer, was incorporated into the blend. In UHPC, the pivotal constituents are fine powders and fine aggregates, and their proportions significantly influence the material’s properties. Table 3 summarizes the optimized mix composition employed in this study. Furthermore, Table 4 provides a comprehensive overview of the varying cement replacement levels, specifically 0%, 5%, 10%, and 20%, achieved by incorporating GP into the mix.

#### 2.2.3. Flexural Test

**Figure 11:**
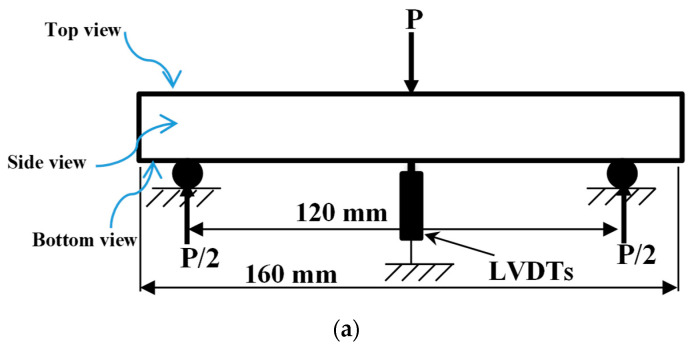

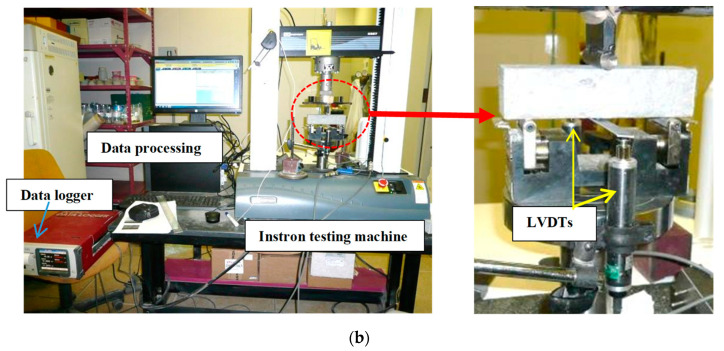
The flexural strength test: (**a**) specimen and (**b**) setup.

In this study, the flexural assessments of the UHPC mixes were performed in compliance with ASTM C1609 guidelines [42]. To measure sample deflection during these tests, a pair of linear variable differential transformers (LVDTs, model FDP 50A, featuring a sensitivity of 300 × 10^−6^ strain/mm; by TML–Tokyo Sokki, Japan) were positioned beneath the test specimens, as shown in Figure 11. A number of three concrete prisms, with dimensions of 75 × 75 × 225 mm^3^, served as the test samples for evaluating three-point flexural strength. These tests were conducted using a 30 kN Instron testing apparatus (Model 3367, Frank Bacon Machinery Sales Co., Warren, MI, USA) at a 0.2 mm/min loading velocity. Data acquisition was facilitated through a Tokyo Sokki system (model TDS-630), which was interfaced with the LVDTs to monitor deflection continuously. The reported outcomes are the mean values derived from three individual test specimens.

## 3. Results and Discussion

### 3.1. Strain Hardening Properties of the PET Fibers

**Figure 12:**
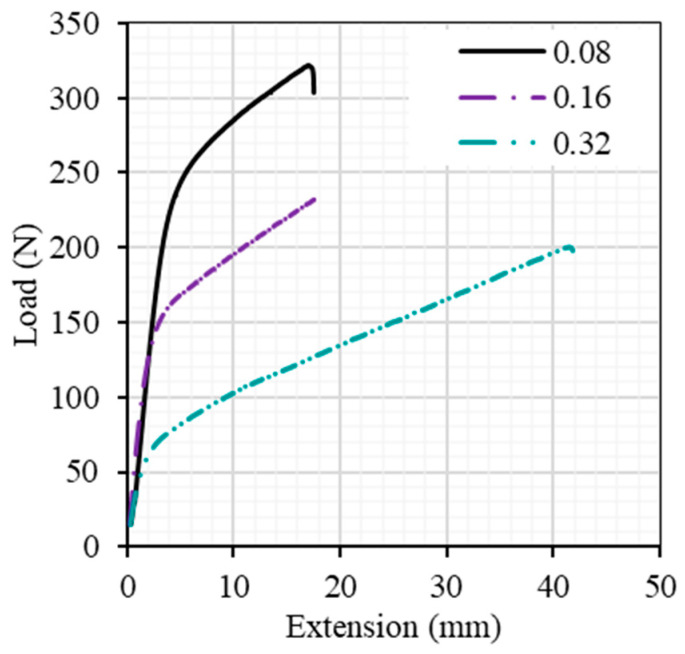
Load–extension relationship at different displacement rates of plastic bottle strips.

In this study, we systematically examined various plastic bottle strips, as visually depicted in Figure 9, meticulously prepared and subjected to testing across a range of strain rates (0.08, 0.16, and 0.32 mm/min). It was observed that the maximum tensile stress exhibited a discernible reduction, in direct correlation with the strain rate, as demonstrated in Figure 12. It is essential to emphasize that this conclusion warrants further comprehensive investigation to elucidate the underlying mechanisms fully. Figure 12 additionally illustrates that the elastic response of PET strips to the uniaxial load exhibited a notable strain independence (the initial linear response remained consistent across all strain rates). It is noteworthy that this observation agrees with the findings reported by Yokouchi et al. [43], who investigated the iMPact of tensile strain rate on the mechanical properties of pre-oriented PET sheets and noted a strain rate independence in specific structural contexts. In a related context, Dupaix [44] explored the finite strain behavior of PET and identified molecular orientation as the predominant mechanism contributing to its hardening and stiffening. Furthermore, Bastun [45] explored the stress distribution in a broad strip subjected to uniaxial tension and discerned the presence of transverse stress within the material during elastoplastic strains.

### 3.2. Flexural Behavior of UHPC Incorporating PBF

**Figure 13:**
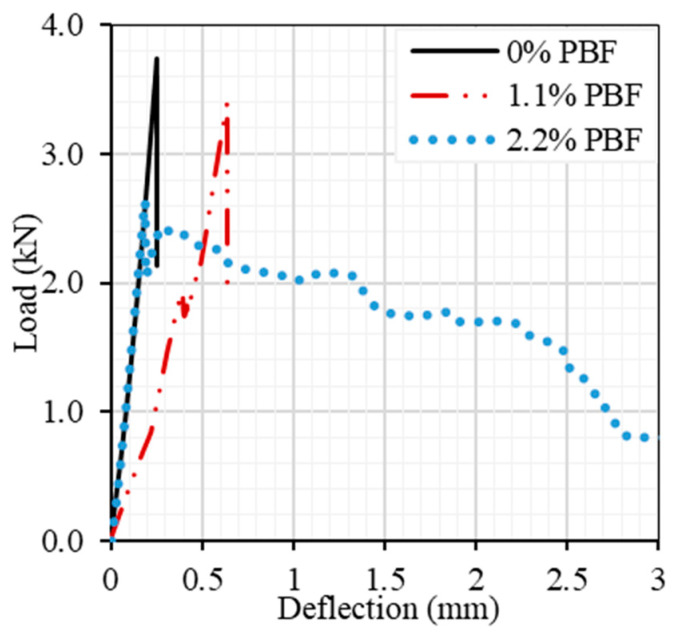
Effect of the presence of PBF on the flexural properties of UHPC.

In this phase of the study, the utilization of PBF as the sole fiber component in the UHPC (Table 5) mix was investigated. This investigation coMPared the flexural properties of mixtures containing 1.1 and 2.2% (vol.) PBF against a control mix (with no PBF) following a 28-day normal curing period. The results, depicted in Figure 13, revealed that introducing 2.2% PBF led to a reduction in ultimate flexural strength, decreasing from approximately 3.4 kN to 2.63 kN, albeit accoMPanied by an increase in ductility.

**Figure 14:**
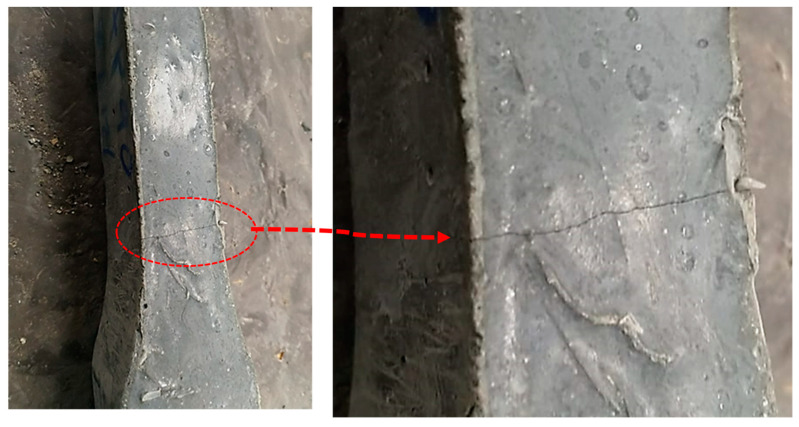
Tested dog bone sample with an induced cross-sectional crack.

**Figure 15:**
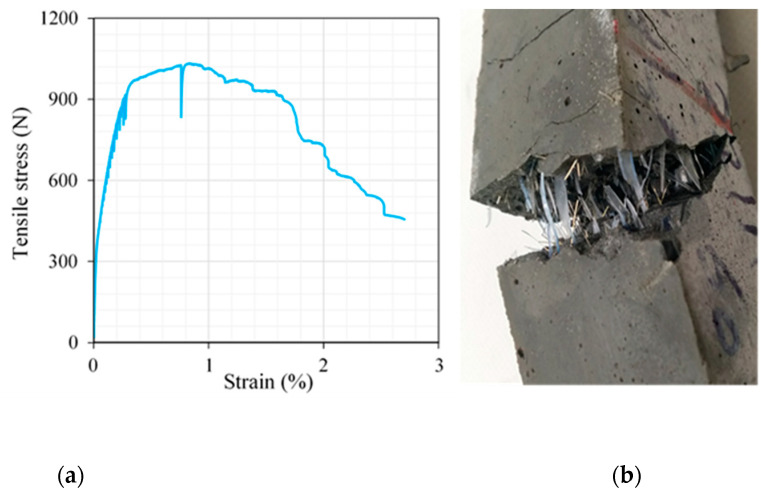
Tensile behavior of UHPC containing 2.2% PBF: (**a**) stress–strain response and (**b**) model of failure.

To gain deeper insights into the performance of UHPC containing 2.2% PBF, we created a specimen with this specific fiber content, including a pre-existing cross-sectional crack, as shown in Figure 14. This test directly assessed how PBF influenced the material’s ability to resist tensile forces. Notably, the specimen exhibited a clear strain-hardening response, as evident in Figure 15. This highlights the remarkable capability of PBF fibers to resist tensile strain effectively. However, it is essential to note that as strain levels reach critical thresholds, the fibers undergo significant elongation, ultimately resulting in a strain-hardening response. To address this phenomenon, we are investigating the hybridization of microsteel fibers with PBF fibers as a potential solution. The incorporation of PET fibers into UHPC has demonstrated a notable enhancement in its flexural properties. In the given context, Alani et al. [46] demonstrated that the incorporation of 1% PET fibers into UHPC led to a substantial increase in the ductility index, accoMPanied by a remarkable enhancement in flexural strength, reaching 30.1 MPa. Similarly, Ali et al. [47] illustrated that the inclusion of 1% PET fibers, distributed in layers, significantly improved the ultimate flexural load. Additionally, Alani et al. [48] observed that the augmentation of PET fibers resulted in an impressive 63.24% increase in the flexural strength of UHPC beams coMPared to their counterparts.

### 3.3. Binary Fiber Hybridization in the Presence of GP

In this phase, we investigated the slump–flow and strength characteristics of UHPC specimens, which contained varying proportions of GP (i.e., 0, 5, 10, 20, 30, and 4%). The assessment was performed after a curing period of 28 days under normal conditions. Additionally, we evaluated the influence of the hybridization of microsteel fibers with fibers derived from PBF. The precise proportions of both the fibers and the GP are listed in Table 5. The optimized quantity of PBF (2.2%, vol.) was employed in the binary hybridization systems (with SFib1 and Sfib2, see Figure 7).

**Figure 16:**
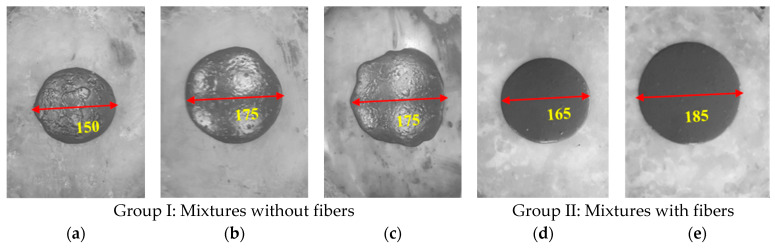
The mini-slump–flow (mm) of (**a**) UP0, (**b**) UP5, (**c**) UP10, (**d**) UP20, and (**e**) UP40.

**Figure 17:**
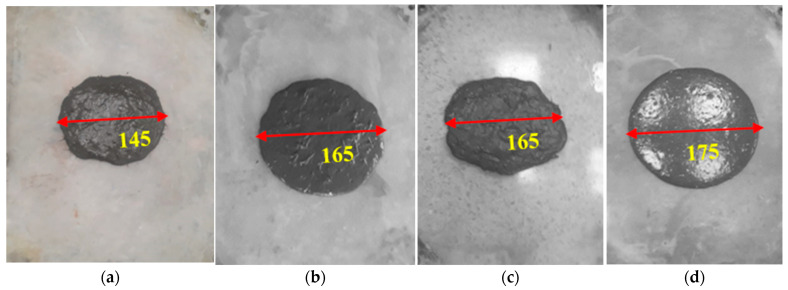
The mini-slump–flow (mm) of (**a**) UP4, (**b**) UP5, (**c**) UP6, and (**d**) UP7.

The flow characteristics of the concrete mixtures were categorized into two distinct groups: (i) without fibers and (ii) with fibers, as visually illustrated in Figure 16a. The first group (UP0–UP40) without fiber content was prepared to investigate the influence of GP substitution levels. The pronounced augmentation effect of GP substitution levels on flowability was readily discernible when examining Figure 16 and Figure 17. Meanwhile, the second group of samples (UP4–UP7) demonstrated that the absence of PBF led to a marked enhancement in flowability, as depicted in Figure 17. It is noteworthy to emphasize that this observed outcome aligns consistently with the findings reported by Ghareeb et al. [49] and Belkadi et al. [50], who similarly observed advantageous iMPacts on the fresh and hardened state properties of concrete. 

**Figure 18:**
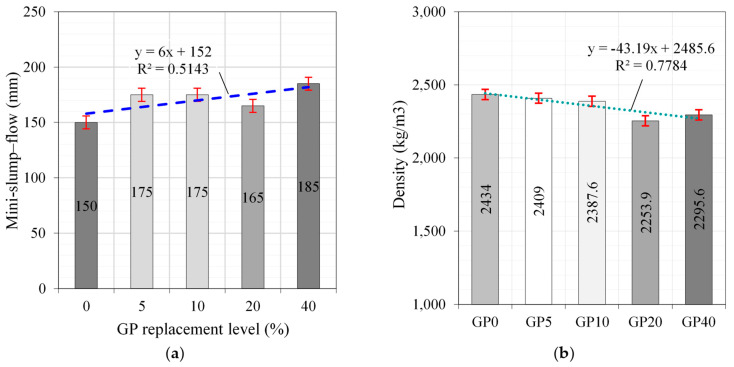
Effect of GP replacement level on (**a**) flowability and (**b**) unit weight.

Furthermore, it is essential to recognize that the inclusion of GP in the mixture led to a notable enhancement in flowability with a gradual reduction in the overall density of the concrete compositions, as illustrated in Figure 18a,b, respectively. The GP inherently had a lower density when coMPared to conventional cementitious materials. Consequently, incorporating GP into the UHPC mix entailed substituting some of the denser components, such as cement, resulting in an overall reduction in the composite’s density [51].

### 3.4. Mechanical Properties of the Studied UHPC Mixtures

#### 3.4.1. Compressive Strength

**Figure 19:**
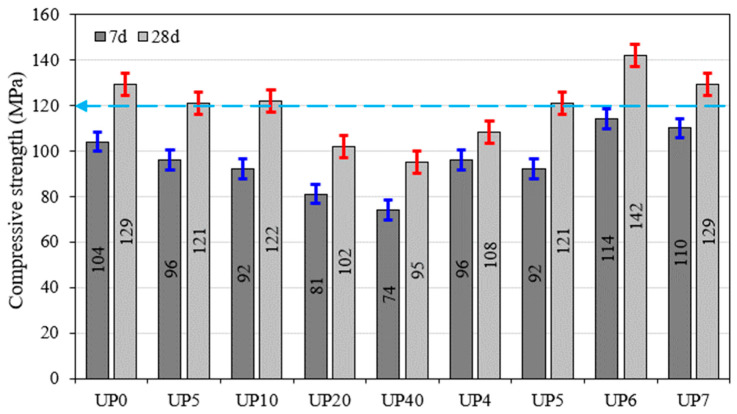
Compressive strength of UHPC mixtures at 28 days.

**Figure 20:**
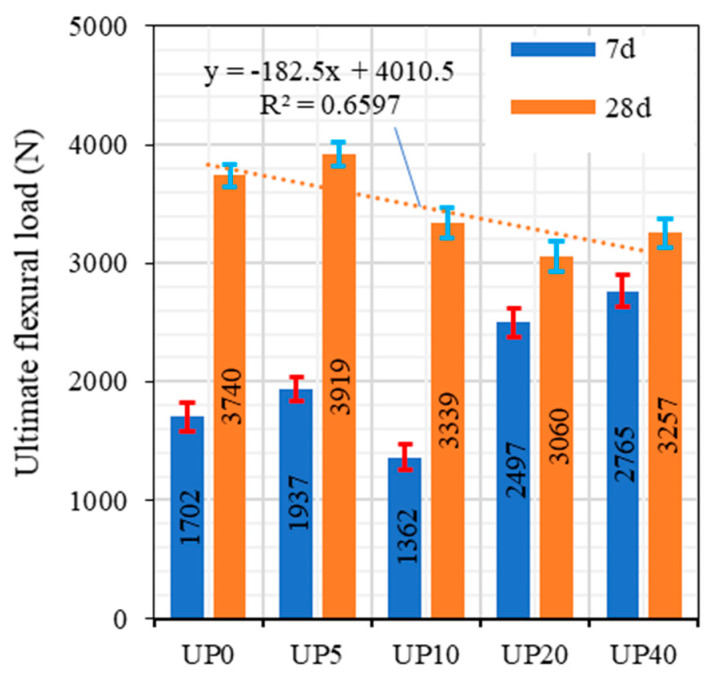
IMPact of the GP content on the ultimate flexural load.

The assessment of compressive strength within the UHPC mixtures (Figure 19) illuminated a clear stratification into two distinct categories. The initial category encoMPassed mixtures devoid of fiber reinforcement, denoted as UP0 through UP40. In contrast, the second category comprised mixtures enriched with fibers, specifically UP4 through UP7. The evolution of compressive strength, examined at the 7-day and 28-day intervals, is meticulously presented in Figure 19. During the 7-day curing period, a consistent pattern emerged: there was a gradual decline in compressive strength as the glass powder (GP) content increased from 0% to 40%. This decline became notably pronounced as the GP content surpassed 10%. In particular, the compressive strength diminished from 104 MPa for the GP-free mixture to 74 MPa for the mixture containing 40% GP. As the curing period extended to 28 days, a similar trend endured, although the UHPC mixtures incorporating 5% and 10% GP exhibited strengths above the widely accepted lower threshold of 120 MPa for UHPC [41,52,53]. The mixture devoid of GP demonstrated the highest compressive strength, registering 129 MPa, while the blend containing 40% GP exhibited the lowest, with a reading of 95 MPa. Notably, these findings align consistently with those reported by Nan et al. [54], who ascertained that replacing cement and silica fume with glass powder can reduce UHPC’s compressive strength. Furthermore, Figure 20 unveils that the most favorable compressive strength outcome was achieved with the UP6 mixture, characterized by 2.2% plastic bottle fibers (PBFs), 1.3% microsteel fiber Sfib1, and a 10% GP content. This observed decline in compressive strength can be attributed to several contributing factors, foremost among them being the lower density of GP coMPared to conventional cementitious materials. This discrepancy in density can result in reduced interparticle bonding within the concrete matrix, weakening the overall structural integrity. Notably, this reduction in compressive strength was particularly pronounced at the 7-day milestone, underscoring the substantial influence of GP on the early-stage strength development of the concrete.

#### 3.4.2. Load Deflection Responses to Flexural Loading

The findings on the flexural characteristics of the concrete mixtures, evaluated after 7 days and 28 days, are graphically depicted in Figure 20. Initially, with a glass powder content of 0%, the maximum flexural load at 7 days was recorded at 1702 N. Notably, introducing a mere 5% glass powder content yielded a considerable increase in flexural load, elevating it to 1937 N. This observation signifies an enhancement in early strength coMPared to the control mixture. However, at a glass powder content of 10%, a discernible reduction in flexural load was apparent, plummeting to 1362 N. The subsequent escalation to 20% glass powder content precipitated a substantial decrease, with the flexural load plummeting to a mere 247 N. The remarkable resurgence in the maximum flexural load when the glass powder content reached 40%, surging impressively to 2765 N, is of particular interest. This nonlinear trend underscores the intricate relationship between glass powder content and concrete’s flexural strength.

Shifting the focus to the 28-day evaluation, in the absence of glass powder (0%), the maximum flexural load stood robustly at 3470 N, signifying the concrete’s continued strength development over time. When 5% glass powder was incorporated, a notable long-term strengthening effect became evident, with the flexural load soaring to 3919 N. Intriguingly, the mixture featuring 10% glass powder, which experienced a dip in strength at 7 days, exhibited a remarkable recovery at 28 days, boasting a flexural load of 3339 N. However, the sample characterized by 20% glass powder content, despite its substantial reduction in strength at 7 days, persisted in displaying lower strength at 28 days, with a load of 3060 N. Notably, the pinnacle of strength at 28 days was attained in the mixture containing 40% glass powder, registering an impressive maximum flexural load of 3257 N.

These findings shed light on the intricate and nonlinear relationship between glass powder content and concrete’s flexural strength. The data suggest that the influence of glass powder on concrete strength is nuanced, and the optimal glass powder content may vary depending on specific applications and curing durations. Further analysis and experimentation are imperative to unraveling these variations’ underlying mechanisms and finetuning glass powder content to meet precise engineering requirements.

It is worth noting that these findings align with previous research. Tagnit-Hamou et al. [55] and Hama et al. [56] underscored that augmenting the glass powder content in UHPC can substantially increase flexural strength. Moreover, Kumar et al. [57] and Soliman and Tagnit-Hamou [58] demonstrated that the optimal glass powder content for maximizing flexural strength typically falls within 10% to 20%. This optimal range strikes a balance, as excessive glass powder content can impede workability and heighten the risk of cracking in concrete formulations.

**Figure 21:**
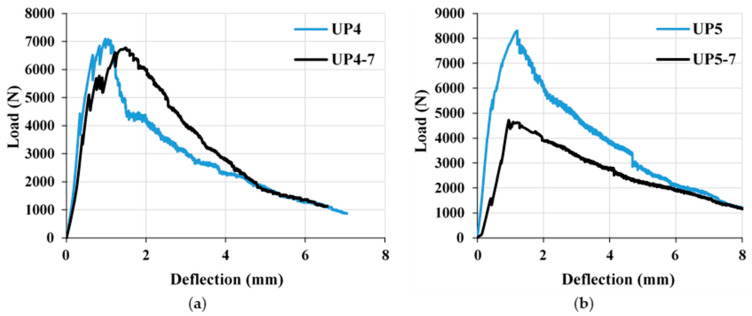

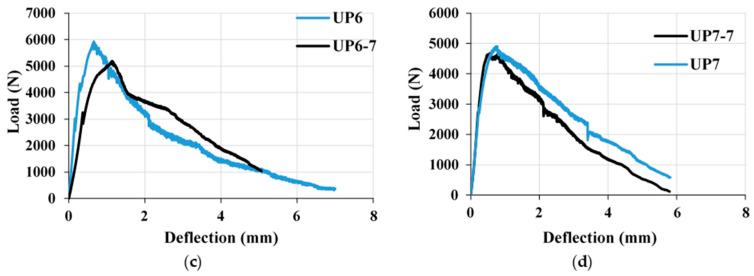
CoMParison of the flexural properties of mixtures (**a**) UP4-7 and UP4, (**b**) UP5-7 and UP5, (**c**) UP6-7 and UP6, and (**d**) UP7-7 and UP7 at curing ages of 7 and 28 days, respectively.

In the formulation of UHPC, we employed an optimal volume-based PBF content of 2.2%, in conjunction with microsteel fibers comprising 1 and 1.3% (Figure 19). The outcomes of these formulations are graphically depicted in Figure 21a,b. The performance data for the mixtures are denoted as UP4–UP7, measured at both 7 and 28 days. Mixtures UP4 and UP5 were formulated with 2.2% PBF and 1% and 1.3% Sfib1, respectively, as indicated in Figure 19. On the contrary, UP6 and UP7 contained 2.2% and 0% PBF, along with 1% and 1.3% Sfib2, respectively, as previously detailed. A comprehensive coMParison of these mixtures at both the 7-day and 28-day intervals is presented in Figure 21c,d. Notably, the results demonstrate that UP5 yielded the most favorable flexural behavior, particularly after the 28-day testing period.

**Figure 22:**
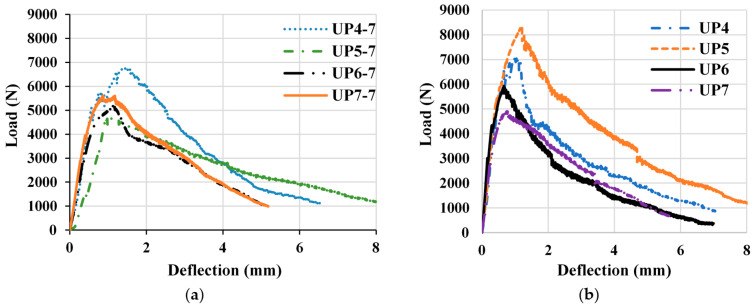
CoMParison of the flexural properties of mixtures UP 4 to UP 7 at a curing age of (**a**) 7 days and (**b**) 28 days.

**Figure 23:**
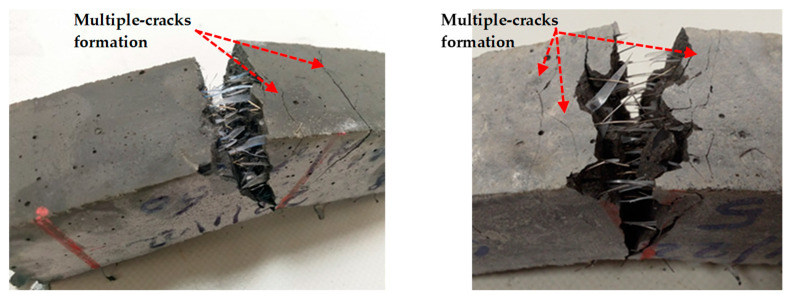
Multiple crack formation due to the strain hardening properties inflicted by the presence of PBF.

The mixtures UP4 and UP5 contained 2.2% PBF and 1 and 1.3% Sfib1, respectively, while UP6 and UP7 had 2.2 and 0% PBF and 1 and 1.3% Sfib2, respectively, as shown in Figure 19. The coMParison among mixtures at 7 and 28 days is shown in Figure 22, respectively. UP5 provided optimal results after 28 days. The incorporation of PBF, chiefly responsible for enhancing the mix’s strain-hardening properties, induced the formation of multiple cracks. These outcomes are visually illustrated in Figure 23. It is notable that the fibers ruptured during the flexural test as an indication of their strong bonding with plastic fibers.

The experimental results provided in this study confirmed the importance of investigating different methodologies for recycling waste materials. The incorporation of glass cullet powder into the cementitious matrix increased its flowability, while the incorporation of plastic fibers reduced it. A balance between the glass cullet powder content and plastic fiber volume can be estimated to balance both effects and end up with improved properties. Ductility is improved when plastic fibers are incorporated, resulting in the formation of multiple cracks that are absent in conventional UHPC. The incorporation of glass cullet within the range of 5 to 10% as a partial replacement of the cement mass was achieved with a corresponding 5 to 10% clinker reduction, a reduced carbon footprint, and a good environmental iMPact. Therefore, the direct iMPact of this approach is accoMPanied by the manufacture of a UHPC mix with a reduced carbon footprint and reduced energy due to a reduction in clinker content and a reduction in the amount of waste. The real-world applications and potential of eco-innovative UHPC in construction projects with the need for elevated ductility and recycling iMPacts are presented in this study.

## 4. Conclusions

This research endeavor strives to amalgamate two interrelated yet often independently explored domains: the utilization of glass powder (GP) and thermally processed PET fibers, denoted here as PBFs, in developing construction materials. The overarching objective is to formulate an ultra-high-performance concrete (UHPC) mixture that incorporates both GP and PBF, aligning seamlessly with contemporary engineering standards and requirements. A distinguishing hallmark of this innovative approach is the incorporation of specialized microsteel fibers, meticulously selected to augment the material’s mechanical attributes. This holistic strategy not only elevates the resulting composite’s mechanical properties but also bolsters its structural resilience across diverse stress scenarios. By harnessing this comprehensive methodology, this research endeavors to present an advanced and versatile concrete blend that can potentially establish new benchmarks within the industry. This groundbreaking mixture is anticipated to redefine the landscape of material quality by ushering in elevated standards in areas encoMPassing strength, durability, and ductility. The implications of this research extend to potential transformative iMPacts within the construction sector, fostering a new era of high-performance construction materials. Using glass powder and plastic bottle fibers presents a promising and cost-effective solution to addressing the environmental iMPact posed by hazardous glass waste and plastic bottles. The findings derived from this comprehensive study led to the following significant conclusions:

(1) An optimal PBF concentration exists, beyond which the influence of fibers on deflection becomes negligible, reaching approximately 0.64 mm. Specifically, at a PBF content of 2.2%, deflection was extended to 2.8 mm.
(2) Plastic bottle fibers exhibit remarkable strain-hardening characteristics, as demonstrated through the initiation of cross-sectional cracks for evaluation.
(3) Varied replacement levels, ranging from 0% to 40% of glass powder (GP), were examined across different mixtures. Our results highlight that a 10% GP replacement level is the optimal choice. Including 10% GP significantly enhances the flexural properties of UHPC mixtures containing microsteel fibers.
(4) In binary hybridization involving PBF and microsteel fibers, we observed a pronounced iMPact on flowability; specifically, with a constant content of 2.2% PBF, the addition of microsteel fibers with an aspect ratio of 100 led to substantial flowability changes coMPared to those with an aspect ratio of 65, where flowability exhibited only slight variations.
(5) The strain rate emerged as a critical factor influencing ultimate tensile stress, with a notable reduction observed as the strain rate decreased.
(6) These findings collectively contribute to understanding the potential advantages and optimal parameters for incorporating glass powder and plastic bottle fibers in UHPC formulations, addressing both environmental concerns and material performance enhancement.

## Figures and Tables

**Table 1:** Chemical analysis of fine powders.

Oxide (%)	GP	FA	SF	PC
CaO	0.34	1.32	2.19	64.14
MgO	4.14	0.31	1.31	0.71
SiO_2_	68.83	55.23	86.20	20.41
Fe_2_O_3_	0.08	10.17	3.79	4.1
Al_2_O_3_	0.36	25.95	0.49	5.32
Na_2_Oeq	20.45	0.86	2.80	0.1
SO_3_	0.24	0.18	0.74	2.44
LOI	5.45	5	2.48	2.18
D50 (µm)	10	11	0.226	10

**Table 2:** Physical properties of microsteel fibers.

Size Group (S)	Sfib1	Sfib2
Aspect ratio	67	83.3
Length (mm)	13	30
Measured diameter (µm)	193	360
Reported diameter (µm)	200	300
Reported aspect ratio	65	100
Density (g/cm^3^)	7.85	7.85

**Table 3:** Mix composition (in kg/m^3^) of the UHPC.

C	SF	FA	RS	W	SP
1107	236	65	633	210	39

**Table 4:** Mix identifications.

Mix ID	GP (%)
GP00	0
GP05	5
GP10	10
GP20	20

**Table 5:** Mix composition (%) of advanced UHPC mixtures with PBF.

Mix ID	PBF	Sfib1	Sfib2	GP
UP0	–	–	–	0
UP5	–	–	–	5
UP10	–	–	–	10
UP20	–	–	–	20
UP40	–	–	–	40
UP4	2.2	–	1	10
UP5	2.2	–	1.3	10
UP6	2.2	1.3	–	10
UP7	–	1.3	–	10

## Data Availability

All data are contained within this article.
